# Case Report: network physiology markers of inter-muscular interactions indicate reversal of age decline with exercise training

**DOI:** 10.3389/fnetp.2025.1686723

**Published:** 2025-11-07

**Authors:** Sergi Garcia-Retortillo, Óscar Abenza, Ladda Thiamwong, Rui Xie, Michelle Gordon, Plamen Ch. Ivanov, Tina E. Brinkley

**Affiliations:** 1 College of Nursing, University of Central Florida, Orlando, FL, United States; 2 Department of Health and Exercise Science, Wake Forest University, Winston-Salem, NC, United States; 3 Complex Systems in Sport, INEFC University of Barcelona, Barcelona, Spain; 4 Faculty of Medicine and Health Sciences, University of Barcelona, Barcelona, Spain; 5 Sticht Center for Healthy Aging and Alzheimer’s Prevention, Wake Forest University School of Medicine, Winston-Salem, NC, United States; 6 Keck Laboratory for Network Physiology, Department of Physics, Boston University, Boston, MA, United States; 7 Department of Neurosurgery, Boston University Chobanian & Avedisian School of Medicine, Boston, MA, United States; 8 Institute of Biophysics and Biomedical Engineering, Bulgarian Academy of Sciences, Sofia, Bulgaria

**Keywords:** aging, network physiology, complex systems, intermuscular coordination, skeletal muscles, electromyography, case report

## Abstract

Aging is associated with a decline in inter-muscular coordination and overall functional capacity. While the benefits of exercise on individual physiological systems are well established, it remains unclear whether regular training can also enhance inter-muscular network interactions and counteract age-related decline. Using a Network Physiology approach, this Case Report investigates the effects of a home-based exercise program on inter-muscular coordination in two older adults. Two older adults (aged 69 and 73) completed a 12-week program that included twice-weekly virtual group sessions, and one weekly session of moderate-intensity aerobic exercise (30 min). Before and after the intervention, participants underwent a maximal cardiopulmonary exercise test (CPET) on a motorized treadmill. During the CPET, surface electromyography (EMG) was recorded from the left and right rectus femoris and biceps femoris. Inter-muscular coordination was quantified using the Amplitude-Amplitude Cross-Frequency Coupling (ACFC) method. Ten time series of EMG band power were extracted for each muscle, representing distinct neuromuscular processes. Pearson’s cross-correlation was then computed for each pair of EMG band power time series across all muscles. Pre-Intervention, both participants showed low overall link strength across all sub-networks. Post-Intervention, there was a pronounced (∼400%) increase in average link strength across all sub-networks in both participants, primarily reflecting enhanced synchronization between distinct frequency bands across the rectus femoris and biceps femoris. These preliminary findings suggest that structured exercise may enhance inter-muscular network coordination in older adults. ACFC-derived network measures offer a promising tool for detecting early age-related decline and evaluating neuromuscular adaptations to exercise interventions.

## 1 Introduction

Assessing inter-muscular coordination in older adults is essential as it directly influences an individual’s capacity for independent functioning, injury prevention, and active participation in daily activities (Engel-Yeger, 2020). Understanding not only the function of individual muscles but also their role within a global inter-muscular network, is crucial for developing effective exercise intervention strategies to improve function and quality of life in older adults (Balagué et al., 2020). However, the impact of aging on inter-muscular network coordination, as well as the potential for structured exercise programs to modulate these network interactions in older adults remain largely unexplored. Here we hypothesize that regular home-based exercise in older adults can enhance inter-muscular network coordination, counteract, and even reverse adverse effects of age-related decline.

Inter-muscular coordination is defined as the level of synchronization between different muscles, contributing to the creation of efficient movement patterns (Garcia-retortillo and Ivanov, 2022; Prilutsky, 2000). It goes beyond simply turning muscles on or off, and encompasses a precise synchronization with specific timing and degree of activation (Comaduran Marquez et al., 2018) that leads to synergetic functions among various muscles during exercise and daily activities. Such fine-tuned synchronization arises from the complex structure of skeletal muscles. A commonly utilized system output to assess inter-muscular coordination is surface electromyography (EMG) (Farina et al., 2004; Gohel and Mehendale, 2020), a non-invasive measure that provides valuable insights into muscle activation and dynamics during movement (Garcia-Retortillo et al., 2023; Merletti and Farina, 2016). Building on the theoretical framework of Network Physiology, which focuses on how functions, states and behaviors at the organism level emerge from integrated networks of diverse physiological and organ systems (Bartsch et al., 2015; Bartsch and Ivanov, 2014; Ivanov, 2021), a novel network-based method—Amplitude-Amplitude Cross-Frequency Coupling (ACFC)—has been recently proposed to quantify inter-muscular network interactions of EMG spectral power time series across distinct muscles (Garcia-Retortillo et al., 2023; Garcia-retortillo and Ivanov, 2022). Recent studies applying the ACFC method have demonstrated increased network stratification in response to fatigue, significant male/female differences in network organization during exercise, and an overall reduction in the degree of inter-muscular and cardio-muscular coordination in older adults (Garcia-Retortillo et al., 2024; Garcia-Retortillo and Ivanov, 2024; Abenza et al., 2025a). These findings suggest that as individuals age, the global inter-muscular network becomes less flexible and adaptable, hindering its ability to respond and reorganize effectively with fatigue and other stimuli.

The effects of regular exercise in minimizing age-related loss of muscle mass (sarcopenia) and power (dynapenia), as well as overall neuromuscular function are well established (Allen et al., 2021). For instance, regular exercise training has been shown to preserve muscle mass with adult aging (Crane et al., 2013; Zampieri et al., 2015), and to benefit the central nervous system (CNS), by preserving cortical brain volume and neurotransmission (Vecchio et al., 2018). However, whether regular exercise can also improve inter-muscular coordination in older adults remains an open question—no studies have directly assessed the impact of exercise on inter-muscular coordination using quantitative dynamic network-based approaches and biomarkers. This gap is critical, as the described age-related impairments in muscle mass and neuromuscular function may be causes, consequences, or both, of a decline in inter-muscular coordination (Garcia-Retortillo et al., 2024). Within the framework of Network Physiology (Ivanov et al., 2016; Ivanov et al., 2017; Liu et al., 2015), and particularly its extension to Network Physiology of Exercise (Balagué et al., 2020; Balagué et al., 2022a; Balagué et al., 2022b), loss of network connectivity or overexpressed synchronization among neuromuscular components and processes plays a key role in functional decline.

Utilizing a Network Physiology approach, the aim of this Case Report is to investigate the effects of a home-based exercise program on inter-muscular coordination in older adults. We observed that exercise intervention positively impacted inter-muscular coordination as evidenced by marked reorganization of the inter-muscular network, bringing network structure and dynamics closer to healthy younger adults. The results of this case study may help inform larger longitudinal and clinical trials designed to comprehensively assess the impact of structured exercise programs on physiological networks in aging populations.

## 2 Methods

### 2.1 Participants

Two older adults (white males, 67 and 65 years, BMI 28.9 and 32.1 kg/m^2^; self-reported knee osteoarthritis) participated in the study. Participants were selected based on inclusion criteria: age (60–80 years); residence in rural area or socioeconomically disadvantaged neighborhood; with history of cardiovascular events; sedentary status with <150 min/wk of moderate-to-vigorous aerobic or resistance exercise in past month; access to smartphone, tablet or computer; internet/Wi-Fi. Exclusion criteria: evidence of cardiac ischemia, congestive heart failure, cognitive impairment or dementia, clinical depression, kidney, neurologic or musculoskeletal disease. The experimental protocol was approved by the Wake Forest University School of Medicine Institutional Review Board, and was conducted in accordance with the Helsinki Declaration. All participants provided signed informed consent after reading and understanding the study details and associated risks.

### 2.2 Study design, test protocol and exercise intervention

Study participants completed a 12-week program consisting of virtual exercise training led by a certified trainer (2 times/week). Each exercise class lasted 45 min and focused on resistance training; it also incorporated stretching, balance, and dual-task exercises. Each week participants were encouraged to complete a third exercise session (>30 min of moderate-intensity aerobic activity) on their own.

To assess cardiorespiratory fitness, a progressive cardiopulmonary exercise test (CPET) was conducted to exhaustion on a motorized treadmill using a ramp protocol (Nicklas et al., 2019). The test included a 5-min warm-up walking at a self-selected speed at a zero incline, followed by the gradually increased/steepened incline to mimic walking uphill. Heart rate was measured continuously using 12-lead electrocardiography, and ventilatory and gas exchange responses were measured on a breath-by-breath basis throughout the test using a computerized system (MGC Diagnostics Ultima system, Minneapolis, MN) to quantify the peak volume of oxygen consumption (VO_2_peak). Participants were asked to give a maximal exhaustive effort confirmed objectively by: respiratory exchange ratio >1.05; perceived exertion rating >17; >90% age-predicted maximal heart rate, consistent with American College of Sports Medicine guidelines (American College of Sports Medicine, 2006). The test was terminated when participants reached volitional exhaustion despite verbal encouragement. As a standard measure of cardiorespiratory fitness, VO_2_peak is presented here as an independent objective parameter of performance, and to contextualize our findings of changes in inter-muscular coordination networks within the overall physiological response to exercise.

### 2.3 EMG acquisition and EMG signal processing

To assess inter-muscular coordination, EMG was acquired during the CPET following SENIAM guidelines (Hermens et al., 2000). EMG signals were simultaneously recorded throughout the entire protocol: left and right rectus femoris (RF-Left, RF-Right), left and right biceps femoris (BF-Left, BF-Right). The exact location of the surface electrodes (Meditrace Foam, E.G., 200, Danlee Medical Products Inc., Syracuse, United States) placement on each muscle was carried out according to SENIAM recommendations. Data were recorded using Biopac MP150 (Biopac Systems Inc., Goleta, CA, United States) and processed using Matlab (Mathworks, Natik, MA, United States). Raw signals were sampled at 2000 Hz, band-pass filtered between 40–250 Hz to reduce low frequency artifacts and motion during running (Papagiannis et al., 2019), and Notch filter at 60 Hz (1 Hz width) to eliminate grid interference. Note that although the power line noise at 60 Hz was effectively removed using the notch filter, no significant noise was detected at the harmonics of 60 Hz (120 Hz and 180 Hz) in the original sEMG data. As a result, we retained these harmonics within the frequency bands F5 and F8, as their removal was deemed unnecessary for the preservation of physiologically relevant information.

### 2.4 Amplitude-Amplitude Cross-Frequency Coupling (ACFC) analyses

**FIGURE 1:**
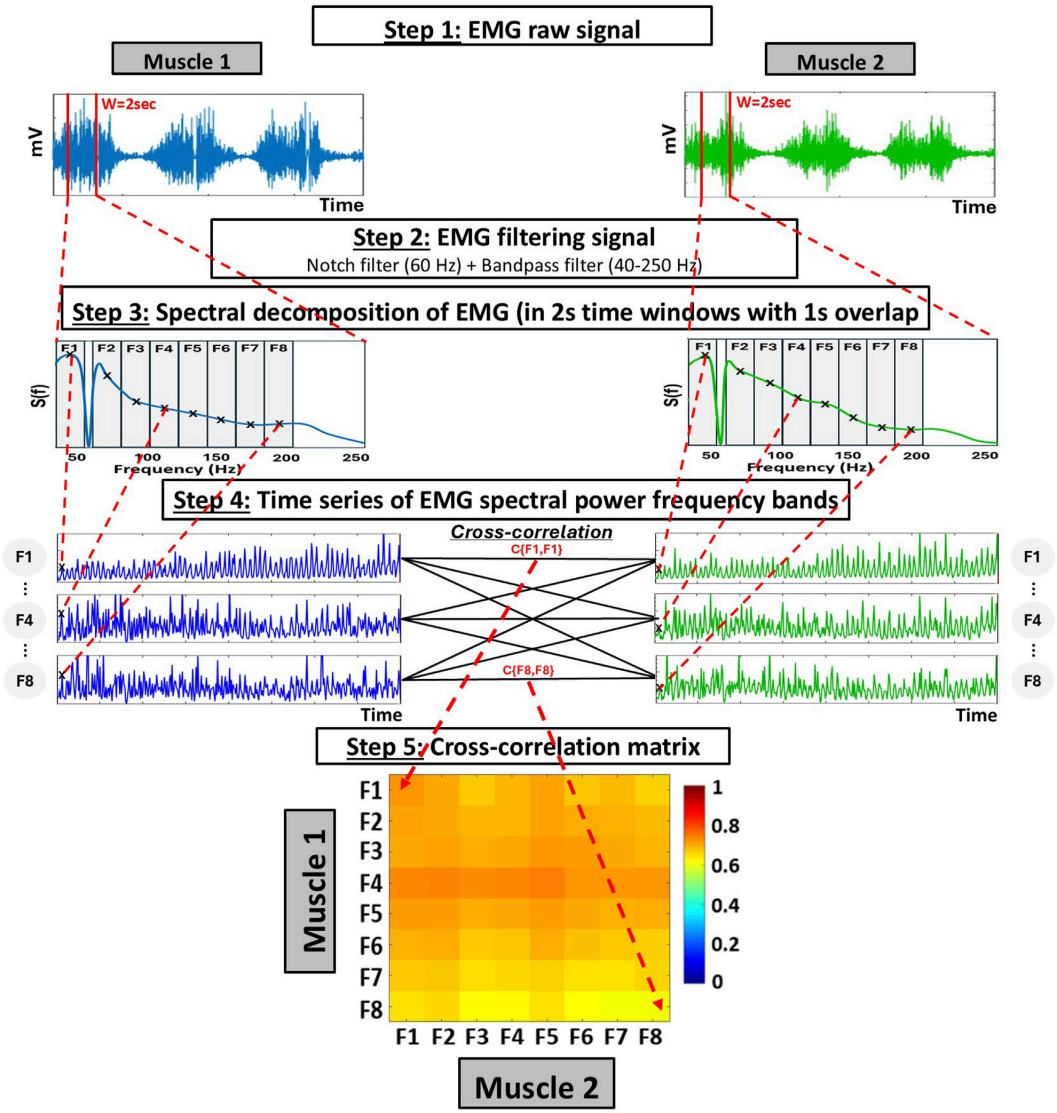
Schematic diagram representing the analyses for the global measures of inter-muscular coordination using the cross-frequency amplitude-amplitude coupling (ACFC) method. Step 1: Raw EMG signals are obtained from two different muscles over the same period. Step 2: Each signal is filtered using a 60 Hz Notch filter to eliminate power line interference and a bandpass filter (40–250 Hz). Step 3: Spectral decomposition is performed on each 2-s time window (with 1-s overlap), using 0.5 Hz bins within the 40–250 Hz range. To capture the specific contribution of different frequency bands, the resulting spectrum is divided into eight frequency bands of equal width (19.5 Hz), and the power within each frequency band is summed to obtain a single value per frequency band and window. Step 4: This process generates a time series of spectral power for each frequency band, sampled at 1-s intervals (i.e., 1s resolution). Bivariate equal-time Pearson’s cross-correlation are then computed at zero lag (no delay) between all combinations of frequency band time series from the two muscles. Step 5: This results in a 8 × 8 cross-correlation matrix (Ci,j) representing the coupling among frequency bands between the two muscles.

The main steps involved in the ACFC method to quantify inter-muscular interactions during the CPET, for both Pre- and Post-Intervention are shown in Figure 1. For detailed explanation of the methodology, see (Garcia-Retortillo et al., 2023; 2024; Garcia-Retortillo and Ivanov, 2022; Garcia-Retortillo and Ivanov, 2024; Abenza et al., 2025a).

#### 2.4.1 Spectral decomposition

EMG signals were segmented into 2-s window with 1-s overlap across the entire exercise duration. Within each 2-s time window: (i) the spectral power $\tilde{S}$ _*δ(f)*_ *(t*_*i*_ *)* was extracted from each EMG signal (based on the discrete Fourier transform), and (ii) $\tilde{S}$ _*δ(f)*_ *(t*_*i*_ *)* values were obtained in bins of 0.5 Hz within the range 10–250 Hz, resulting in N = 480 data points for each window. To calculate the spectral power within each 2-s time windows, we used the “*pwelch”* function in MATLAB, which estimates the power spectral density of the EMG signal through Welch’s method. The “*pwelch*” function applies by default a Hamming window of 4000 samples corresponding to the analysis window length used in our study (2-s time windows). Spectral power values were calculated in each 2-s window with step of 1-s, thus, generating a time series with 1-s resolution for each predefined frequency band [F1, … ,F8] (see detailed description in Garcia-Retortillo et al., 2023; 2024; Garcia-Retortillo and Ivanov, 2022; Garcia-Retortillo and Ivanov, 2024; Abenza et al., 2025a). Unlike the conventional use of the Welch estimator—which averages across multiple sub-segments—here we applied it using a single segment per window, so no averaging was performed. Under these conditions, “*pwelch*” operates as a single-window FFT with a Hamming window, which is identical to an ‘STFT’ (short-time Fourier Transform) implemented with the same parameters.

To probe specific contributions from different frequency bands *F*_*i*_ to the EMG spectral power within each 2-s window, we consider 8 frequency bands with equal width of 19.5 Hz: F1 = [40–59.5Hz], F2 = [60.5–80Hz], F3 = [80.5–100Hz], F4 = [100.5–120 Hz], F5 = [120.5–140 Hz], F6 = [140.5–160 Hz], F7 = [160.5–180 Hz], and F8 = [180.5–200Hz]. These bands potentially reflect the activity of different muscle fiber/motor unit types with distinct firing rates, among other factors (Garcia-Retortillo et al., 2020; Garcia-Retortillo and Ivanov, 2022).

Next, the sum of the power $\tilde{S}\left(f\right)$ across all frequency bins (0.5 Hz width) within each frequency band (19.5 Hz width) was calculated as $\tilde{S}\left(f\right)$ : = $\sum_{i=1}^{n}S($ *ƒ*_*i*_ *)*, where *ƒ*_*i*_ represents all n = 39 frequency bins in each frequency band *F*_*i*_. This yielded eight time series of EMG band power $\tilde{S}$ _*δ(f)*_ *(t*_*i*_ *)* with 1-s resolution for each muscle, representing the dynamics of activity rhythms in EMG.

The obtained time series of EMG spectral power for each band *F*_*i*_ were then normalized to zero mean and unit standard deviation. These normalized time reflect the micro-architecture (1-s resolution) of synchronous modulation in the amplitude of muscle activation, and allow to track variations in network interactions between EMG frequency bands *F*_*i*_ throughout the entire exercise and to assess pre- and post-intervention effects.

#### 2.4.2 Cross-correlations between time series of EMG spectral power in different frequency bands

**FIGURE 2:**
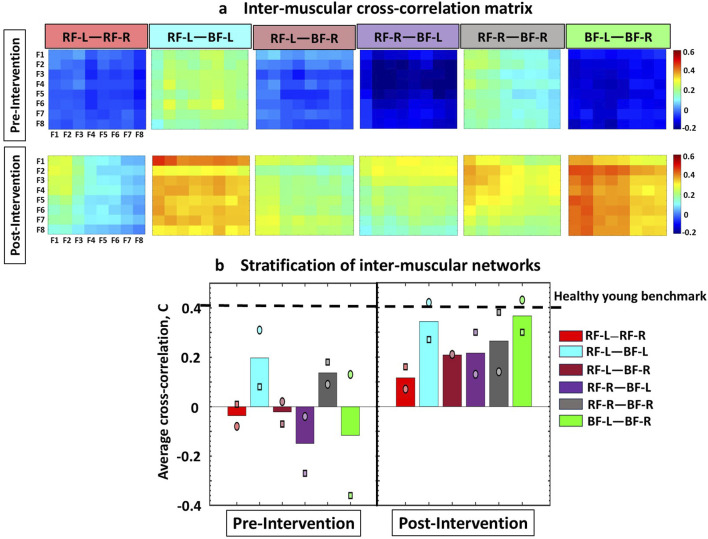
Cross-correlation matrices illustrating inter-muscular interactions between EMG rhythms during Cardio-Pulmonary Exercise Testing (CPET), comparing Pre- and Post-Intervention conditions. **(a)** The inter-muscular cross-correlation matrices represent the group-average cross-correlations for Participants 1 and 2, between the spectral power among eight frequency bands for all muscle pairs. Each matrix represents a sub-network in inter-muscular cross-correlations for each pair of muscles. All frequency bands have equal widths of 19.5 Hz, falling within intervals from 40–59.5 Hz and 60.5–200 Hz. In the matrices, rows represent the spectral power of the Fi frequency bands from the first muscle, while columns represent the spectral power of the Fi frequency bands from the second muscle. The colors in the vertical bars represent the strength of the links between corresponding frequency bands, based on the cross-correlation value C{Fi, Fj}. All sub-networks in both Participants 1 and 2 exhibited higher link strength post-intervention compared to pre-intervention. **(b)** Bar plots illustrate the stratification of global cross-correlation of the inter-muscular sub-networks (obtained from matrices in Figure 2a). Each bar represents the average link strength for each sub-network across Participant 1 and 2, with individual participant values indicated by different symbols (circle for Participant 1, and square for Participant 2). Horizontal line indicates benchmark network connectivity derived in healthy young adults reported in the literature and derived from different exercise protocol using the same ACFC method (Garcia-Retortillo et al., 2023; 2024; Garcia-Retortillo and Ivanov, 2022; Garcia-Retortillo and Ivanov, 2024; Abenza et al., 2025a).

For each muscle pair, we computed the bivariate equal-time Pearson’s cross-correlation for all pairs of time series representing EMG spectral power $\tilde{S}$ _*δ(f)*_ *(t*_*i*_ *)* in the frequency bands *F*_*i*_, where i = 1, … ,8. This results in 8 × 8 = 64 cross-correlation values *C*_*i*_ *,*_*j*_ for each pair of muscles, as shown in the inter-muscular cross-correlation matrices (Figure 2a). Each *C*_*i*_ *,*_*j*_ quantifies the degree of synchronization of EMG frequency band *F*_*i*_ from one muscle with the frequency band *F*_*j*_ from another muscle, ranging from *C*_*i*_ *,*_*j*_ = −1 (anti-correlated) to *C*_*i*_ *,*_*j*_ = 1 (positively correlated), where *C*_*i*_ *,*_*j*_ = 0 indicating no linear relationship.

#### 2.4.3 Inter-muscular cross-correlation matrices

The group-averaged cross-correlation matrices illustrate the pairwise correlation between the eight frequency bands *F*_*i*_ of one muscle and the corresponding bands derived from another muscle (i.e., 6 distinct muscle pairs) during the test (Figure 2a). Each matrix element represents the group-averaged cross-correlation calculated across the two participants in the group, resulting in 64 elements per matrix (cross-correlation coefficients) that represent the interactions for each pair of frequency bands in the muscle pairs.

#### 2.4.4 Inter-muscular interaction networks

**FIGURE 3:**
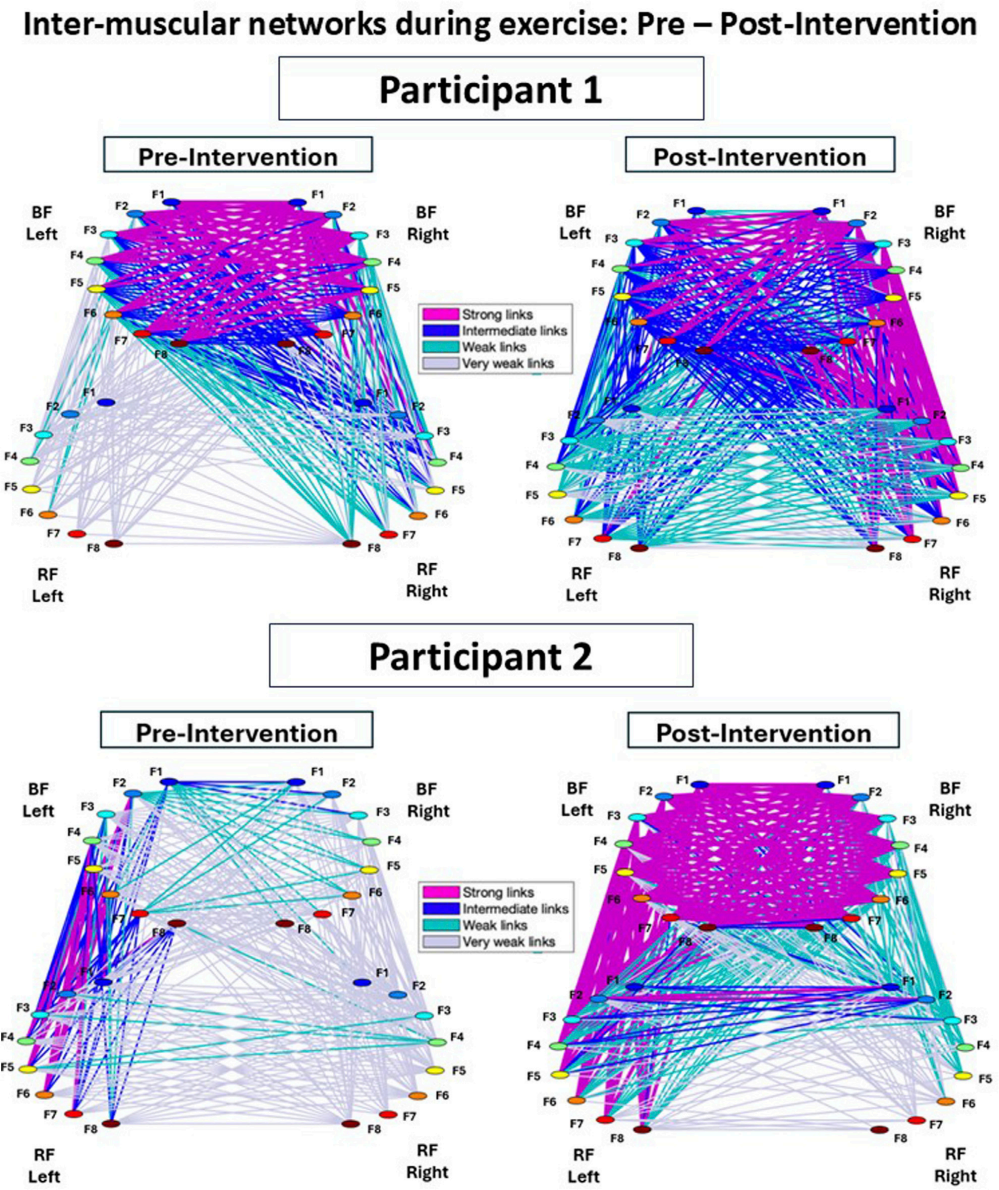
Inter-muscular networks of myoelectrical activity during Cardio-Pulmonary Exercise Testing (CPET), comparing Pre- and Post-Intervention conditions. Network maps are generated from the cross-correlation matrices for each participant, where each link corresponds to the cross-correlation values representing the degree of coupling between distinct muscle rhythms (frequency bands, f_i_). Each muscle is shown as a semicircle with color nodes representing different EMG activity rhythms. The line width and color of the links illustrate the average link strength between frequency bands. In both Participant 1 and 2, the Post-Intervention network showed a marked increase in the strength and number of links for all sub-networks compared to the Pre-Intervention condition.

A multiplex network of sub-networks was obtained to visualize the interactions among all muscle pairs and their hierarchical organization within the network (Figure 3). Each muscle is depicted by a semicircle, where colored nodes represent different frequency bands *F*_*i*_. Network links represent the values of cross-correlation matrix elements *C*_*i*_ *,*_*j*_ in Figure 2a, reflecting the strength of the links between the frequency bands of two different muscles. Average link strength is indicated by line color and width. Links strength is classified as weak links (0.05 < *C*_*i*_ *,*_*j*_ < 0.15; very thin grey lines), intermediate links (0.15 < *C*_*i*_ *,*_*j*_ < 0.25; thin green lines), strong links (0.25 < *C*_*i*_ *,*_*j*_ < 0.35; dark blue thick lines), and very strong links (*C*_*i*_ *,*_*j*_ > 0.35; magenta very thick lines).

**FIGURE 4:**
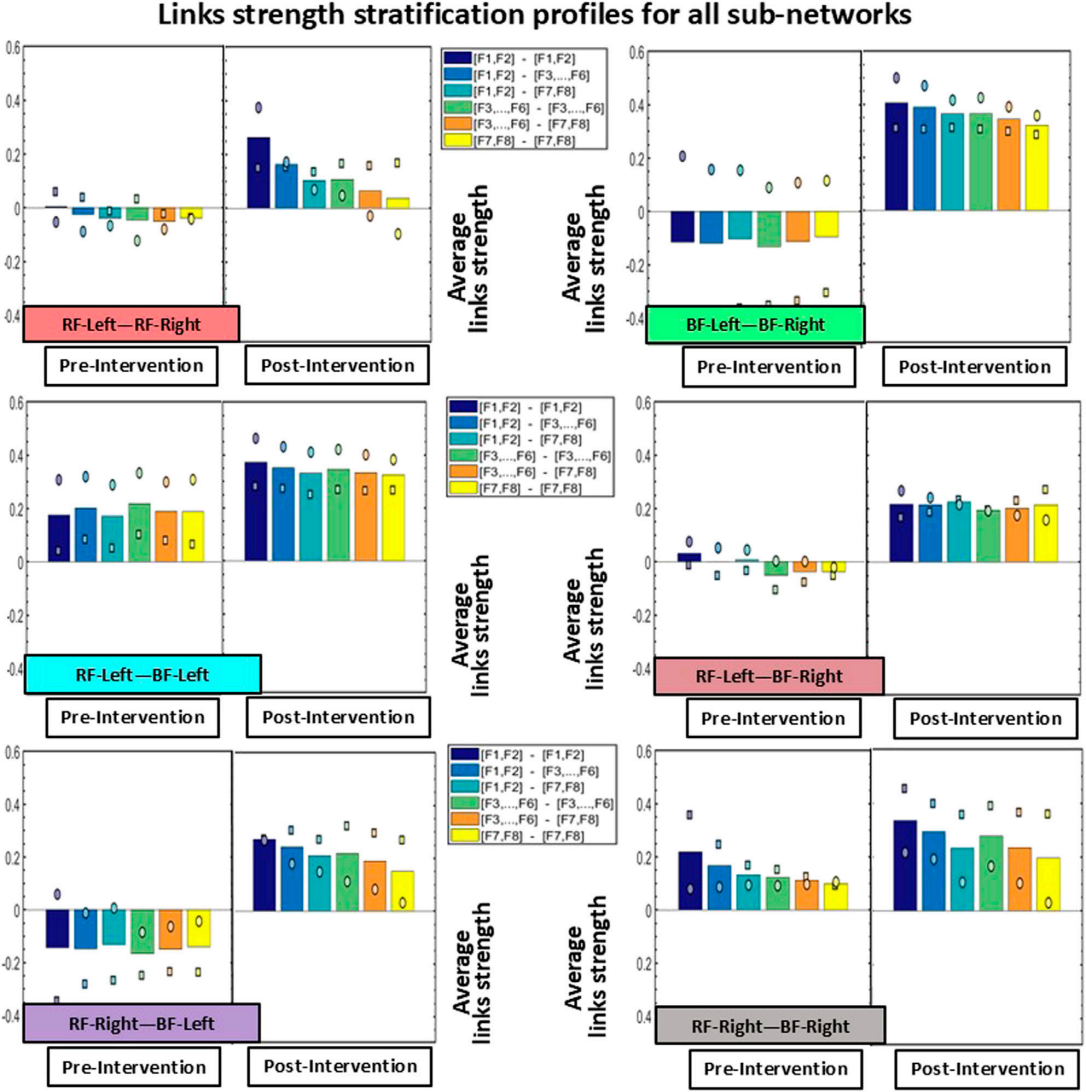
Stratification profiles of links strength for all inter-muscular sub-networks. Each stratification profile consists of six bars, representing the group-averaged links strength of all frequency band cross-correlation modules within a given sub-network. Individual participant values are indicated using distinct symbols (circle for Participant 1, and square for Participant 2). Notably, all modules across all sub-networks showed increased link strength Post-Intervention.

To further dissect the inter-muscular network and to quantify its hierarchical organization, we obtained the stratification profiles for each pair of muscles within the network, hereafter referred to as a ‘sub-network’, based on the group-averaged cross-correlation matrix (Figure 2a). Considering that each sub-network represents the correlations between EMG frequency bands for a given pair of muscles, we analyzed the following network modules of frequency cross-correlation in each sub-network: low-low [(F1-F2)—(F1-F2)], low-intermediate [(F1-F2)—(F3 … F6)], low-high [(F1-F2)—(F7-F8)], intermediate-intermediate [(F3 … F6)—(F3 … F6)], intermediate-high [(F3 … F6)—(F7-F8)], and high-high [(F7-F8)—(F7-F8)] (Figure 4) (see Garcia-Retortillo et al., 2023; 2024; Garcia-Retortillo and Ivanov, 2022; Abenza et al., 2025a for details).

## 3 Results

Exercise performance was assessed by measuring VO_2_peak. The pre-intervention VO_2_peak for Participants 1 and 2 was 20.0 and 16.7 mL/kg/min, respectively. After the 12-week exercise intervention Participant 1 had a VO_2_peak of 17.1 mL/kg/min (change: -2.9 mL/kg/min, 14.5%), whereas Participant 2 had a post-intervention VO_2_peak of 18.4 mL/kg/min (change: +1.7 mL/kg/min, 10.2%).

Figure 2 shows the inter-muscular cross-correlation matrices, illustrating group-averaged interactions from Participants 1 and 2. These matrices represent the correlations between the spectral power of eight frequency bands across all muscle pairs, both before and after the intervention. Overall, the Post-Intervention condition showed higher cross-correlation values among all sub-networks (pairs of muscles) in both participants. In Participant 1 (represented by a circle marker), the Post-Intervention condition resulted in marked increases in link strength between all sub-networks. Specifically, the RF-Left—RF-Right sub-network increased by ∼190%, RF-Left—BF-Left by ∼35%, RF-Left—BF-Right by ∼950%, RF-Right—BF-Left by ∼430%, RF-Right—BF-Right by ∼55%, and BF-Left—BF-Right ∼230%. Participant 2 (represented by a square marker) also showed marked increases in cross-correlation values after the Intervention. In this case, the RF-Left—RF-Right sub-network increased by ∼1500%, RF-Left—BF-Left by ∼240%, RF-Left—BF-Right by ∼400%, RF-Right—BF-Left by ∼210%, RF-Right—BF-Right by ∼110%, and BF-Left—BF-Right by ∼180% (Figure 2b).

Figure 3 shows the inter-muscular networks during the CPET for Participant 1 (top row) and Participant 2 (bottom row), comparing Pre- and Post-Intervention. In the Pre-Intervention condition, Participant 1 exhibited generally weak links across the network, except within the BF-Left—BF-Right sub-network. Similarly, Participant 2 showed weak overall links, with relatively stronger links in the RF-Left—BF-Left sub-network. Post-Intervention, both participants preserved similar hierarchical organization compared to Pre-Intervention; however, the overall link strength and number of links remarkably increased across all sub-networks.

Notably, Participant 1 showed stronger links predominantly on the right side of the network, whereas Participant 2 exhibited stronger links primarily on the left side.

Figure 3 shows the inter-muscular networks during the CPET for Participant 1 (top row) and Participant 2 (bottom row), comparing the Pre- and Post-Intervention conditions. In the Pre-Intervention, Participant 1 presented overall weak links in the entire network, except with the BF-Left—BF-Right sub-network. Participant 2, meanwhile, also presented weak overall links, with some stronger links in the RF-Left—BF-Left sub-network. In Post-Intervention, while both participants exhibited a consistent hierarchical network organization between conditions, the overall links strength as well as the amount of links remarkably increased for all sub-networks. This increment was more pronounced in sub-networks involving the BF (i.e., BF-Left—BF-Right, RF-Left—BF-Left, RF-Left—BF-Right, RF-Right—BF-Left, and RF-Right—BF-Right). Notably, Participant 1 exhibited stronger links predominantly on the right side of the network; in contrast, Participant 2 showed stronger links mostly on the left side of the network, reflecting potential coordination asymmetries.

The stratification profiles of each sub-network, used to quantify the hierarchical organization of the inter-muscular network (Figure 4), revealed substantial differences in cross-correlation values across most modules. Consistent with the patterns observed in Figures 2, 3, the BF-Left—BF-Right sub-network showed the most pronounced increase in link strength in the Post-Intervention condition for both participants. Specifically, Participant 1 (circle marker) exhibited an average increase of approximately 120% across the six modules, while Participant 2 (square marker) showed an even greater increase of approximately 220%. Regarding the other sub-networks, all five showed higher cross-correlation values across all network modules in the Post-Intervention condition for both participants. In Participant 1, links strength increased by approximately 70% in the RF-Left—RF-Right sub-network, ∼25% in RF-Left—BF-Left, ∼90% in RF-Left—BF-Right, ∼145% in RF-Right—BF-Left, and ∼15% in RF-Right—BF-Right. Similarly, Participant 2 exhibited increases for all sub-networks and network modules: ∼95% in RF-Left—RF-Right, ∼75% in RF-Left—BF-Left, ∼130% in RF-Left—BF-Right, ∼190% in RF-Right—BF-Left, and ∼50% in RF-Right—BF-Right.

## 4 Discussion

Using a network-based approach, this Case Report demonstrates the effects of a home-based exercise program on inter-muscular coordination in older adults. The exercise intervention improved inter-muscular coordination, as evidenced by a marked increase in links strength within the inter-muscular network. Notably, these improvements occurred despite no measurable increase in exercise performance, highlighting the sensitivity of inter-muscular coordination as a functional biomarker that may respond to neuromuscular adaptations earlier than traditional performance outcomes. The findings of this case study suggest that regular exercise in older adults could benefit not only individual neuromuscular components (e.g., muscles and muscle fibers, motor units) but also strengthens the dynamic network interactions among them, bringing the network of inter-muscular coordination close to the expected healthy behavior in young adults.

The overall degree of link strength observed Pre-Intervention was low in both Participants across all sub-networks (RF-Left—RF-Right, RF-Left—BF-Left, RF-Left—BF-Right, RF-Right—BF-Left, RF-Right—BF-Right, and BF-Left—BF-Right), indicating a reduced level of synchronization between EMG frequency bands across muscles—which, in turn, suggests a low level of inter-muscular coordination across muscle fibers. These results are consistent with recent studies reporting diminished link strength in inter-muscular networks during exercise in older adults compared to younger individuals (Garcia-Retortillo et al., 2024). This reduction in inter-muscular coordination may be associated with age-related neuromuscular changes, such as sarcopenia, decreased number of larger motor units, reduced synaptic input, instability at the neuromuscular junction, or lower motor unit discharge rates (Hunter et al., 2016; Seidler et al., 2010; Serrien et al., 2000; Sun et al., 2016).

Notably, there was a pronounced increase in average link strength across all muscle sub-networks Post-Intervention in both Participants—driven by enhanced synchronization between distinct EMG frequency bands across the rectus femoris and biceps femoris muscles, indicating improved inter-muscular coordination. These findings are paralleled by previously established effects of regular exercise in mitigating sarcopenia and dynapenia, and in enhancing overall neuromuscular function (Crane et al., 2013; Vecchio et al., 2018; Zampieri et al., 2015), which focused on separated systems but not on their network integration. However, given the nature of this study, we cannot determine whether the observed improvements in inter-muscular coordination are a cause or consequence of increased muscle mass and neuromuscular function—or both (Garcia-Retortillo et al., 2024). Future research should address this question directly, as the Network Physiology framework (Balagué et al., 2020; Balagué et al., 2022b; Bartsch et al., 2015; Bashan et al., 2012; Ivanov, 2021; Ivanov and Bartsch, 2014; Liu et al., 2015) suggests that the loss of connectivity among neuromuscular components may itself contribute to the functional decline of each individual element, and that restoring muscular coordination may result from modulation of regulatory mechanisms affecting directly the dynamics of network links. We interpret between-participant network differences as reflecting inter-individual variability in inter-muscular coordination rather than potential differences in osteoarthritis severity, a hypothesis that should be evaluated in larger, controlled studies.

The observed increase in inter-muscular coordination Post-Intervention was not proportional for all sub-networks, as the BF-Left—BF-Right exhibited a remarkably larger increment in links strength compared to the other sub-networks (Figure 2), showing differentiated response of distinct muscle pairs to exercise and neuromuscular adaptation. This may reflect the growing demand placed on the hamstrings, during incline walking where they contribute significantly to hip extension and propulsion. Such bilateral coordination may become increasingly relevant under fatigue, as symmetrical recruitment of the hamstrings helps counteract compensatory asymmetries and maintain efficient gait mechanics during sustained incline walking. Enhanced synchronization between left and right BF may thus indicate improved neuromuscular efficiency and inter-hemispheric coordination as a result of the exercise program.

Interestingly, the changes in VO_2_peak and inter-muscular coordination followed a similar pattern between participants—Participant 2 exhibited greater increases in both VO_2_peak and inter-muscular coordination following the intervention, whereas Participant 1 showed a reduction in VO_2_peak accompanied by smaller network coordination improvements. This parallel evolution of cardiorespiratory and neuromuscular markers suggests that enhanced inter-muscular coordination may contribute to more efficient oxygen utilization and overall exercise performance, highlighting the potential interplay between coordinative and metabolic adaptations to training. This finding aligns with the idea that coordinative adaptations may precede measurable gains in performance, offering early insights into neuromuscular improvements that traditional metrics may overlook. Notably, gold standard physiological and performance variables (e.g., VO_2_max, heart rate) provide limited information about the nonlinear, dynamic interactions among physiological systems, and they cannot capture the qualitative reconfigurations that occur as these systems coordinate in response to exercise demands and fatigue (Balagué et al., 2016; Garcia-Retortillo et al., 2017; Garcia-Retortillo et al., 2019a; Garcia-Retortillo et al., 2019b; Abenza et al., 2025b). In contrast, inter-muscular coordination metrics may offer a more sensitive window into early functional adaptations, particularly in populations where overt performance gains may take longer to emerge. Therefore, the present findings highlight the potential that network-based approaches and network-derived biomarkers detect subtle neuromuscular adaptations in inter-muscular coordination that precede measurable changes in traditional physiological parameters derived from individual systems.

The small sample size of the study limits the generalizability of the findings. Without statistical inference, we cannot draw generalizable conclusions or confidently attribute changes in inter-muscular coordination to the exercise intervention alone. The primary aim is to demonstrate the methodological feasibility of a dynamic network approach, and provide preliminary evidence that can motivate and guide future larger cohort studies. While the observed improvements in inter-muscular coordination are promising, they should be interpreted as preliminary evidence. Additionally, without a control group, it is not possible to fully attribute the observed changes solely to the exercise intervention. These findings may inform larger longitudinal and clinical trials designed to comprehensively assess the impact of structured exercise programs on physiological networks in aging populations.

In summary, this Case Report provides preliminary evidence that a home-based exercise program may enhance inter-muscular coordination in older adults, as reflected by increased synchronization between EMG frequency bands across leg muscles, and by restoring muscle network dynamics to levels closer to healthy young behavior. While limited by a small sample size and lack of statistical analysis, our empirical findings demonstrate the utility of a dynamic and adaptive network-based approach to characterize neuromuscular adaptations to exercise programs, and provide new regulatory and mechanistic insights on muscle network dynamics.

## Data Availability

The raw data supporting the conclusions of this article will be made available by the authors, without undue reservation.

## References

[B1] Abenza Ó. Garcia-Retortillo S. Vasileva F. Hristovski R. Ivanov P. C. Balagué N. (2025a). Networks of intermuscular coordination distinguish Male and female responses to exercise. Sci. Rep. 2025 15 (1), 33901. 10.1038/S41598-025-08294-7 41028042 PMC12485009

[B2] Abenza Ó. Montull L. Javierre C. Balagué N. (2025b). Cardiorespiratory coordination during exercise recovery: a novel measure for health assessment. Apunts. Educ. Fis. Deport. 159, 1–9. 10.5672/apunts.2014-0983.es.(2025/1).159.01

[B3] Allen M. D. Dalton B. H. Gilmore K. J. McNeil C. J. Doherty T. J. Rice C. L. (2021). Neuroprotective effects of exercise on the aging human neuromuscular system. Exp. Gerontol. 152, 111465. 10.1016/J.EXGER.2021.111465 34224847

[B4] American College of Sports Medicine (2006). ACSM’s guidelines for exercise testing and prescription. 7th ed. Lippincott Williams and Wilkins. Available online at: https://www.scirp.org/reference/referencespapers?referenceid=841435. 10.1249/JSR.0b013e31829a68cf 23851406

[B5] Balagué N. González J. Javierre C. Hristovski R. Aragonés D. Álamo J. (2016). Cardiorespiratory coordination after training and detraining. A principal component analysis approach. Front. Physiology 7 (35), 35. 10.3389/fphys.2016.00035 26903884 PMC4751338

[B6] Balagué N. Hristovski R. Almarcha M. Garcia-Retortillo S. Ivanov P. C. (2020). Network physiology of exercise: vision and perspectives. Front. Physiology 11, 611550. 10.3389/fphys.2020.611550 33362584 PMC7759565

[B7] Balagué N. Garcia-Retortillo S. Hristovski R. Ivanov P. C. (2022a). “From exercise physiology to network physiology of exercise,” in Exercise physiology. Editor Ferraz R. , 11. 10.5772/intechopen.102756

[B8] Balagué N. Hristovski R. Almarcha M. Garcia-Retortillo S. Ivanov P. C. (2022b). Network physiology of exercise: beyond molecular and omics perspectives. Sports Med. - Open 8 (1), 119. 10.1186/s40798-022-00512-0 36138329 PMC9500136

[B9] Bartsch R. Ivanov P. C. (2014). Coexisting forms of coupling and phase-transitions in physiological networks. Commun. Comput. Inf. Sci. 438, 270–287. 10.1007/978-3-319-08672-9

[B10] Bartsch R. Liu K. Bashan A. Ivanov P. C. (2015). Network physiology: how organ systems dynamically interact. PLOS ONE 10 (11), e0142143. 10.1371/JOURNAL.PONE.0142143 26555073 PMC4640580

[B11] Bashan A. Bartsch R. Kantelhardt J. Havlin S. Ivanov P. C. (2012). Network physiology reveals relations between network topology and physiological function. Nat. Commun. 3, 702. 10.1038/ncomms1705 22426223 PMC3518900

[B12] Comaduran Marquez D. Von Tscharner V. Murari K. Nigg B. M. (2018). Development of a multichannel current-EMG system for coherence modulation with visual biofeedback. PLoS ONE 13 (11), e0206871. 10.1371/journal.pone.0206871 30444897 PMC6239290

[B13] Crane P. K. Walker R. Hubbard R. A. Li G. Nathan D. M. Zheng H. (2013). Glucose levels and risk of dementia. Forsch. Komplementarmedizin 20 (5), 540–548. 10.1056/NEJMOA1215740 23924004 PMC3955123

[B14] Engel-Yeger B. (2020). The role of poor motor coordination in predicting adults’ health related quality of life. Res. Dev. Disabil. 103, 103686. 10.1016/J.RIDD.2020.103686 32417632

[B15] Farina D. Merletti R. Enoka R. (2004). The extraction of neural strategies from the surface EMG. J. Appl. Physiology 96 (4), 1486–1495. 10.1152/japplphysiol.01070.2003 15016793

[B17] Garcia-Retortillo S. Ivanov P. C. (2024). Dynamics of cardio-muscular networks in exercise and fatigue. J. Physiology, 1–27. 10.1113/JP286963 39392864

[B16] Garcia-Retortillo S. Ivanov P. C. (2025). Dynamics of cardio-muscular networks in exercise and fatigue. J. Appl. Physiology 603 (18), 5121–5147. 10.3389/fnetp.2022.1059793 39392864

[B18] Garcia-Retortillo S. Javierre C. Hristovski R. Ventura J. Balagué N. (2017). Cardiorespiratory coordination in repeated maximal exercise. Front. Physiology 8 (387), 387. 10.3389/fphys.2017.00387 28638349 PMC5461287

[B19] Garcia-Retortillo S. Gacto M. O’Leary T. Noon M. Hristovski R. Balagué N. (2019a). Cardiorespiratory coordination reveals training-specific physiological adaptations. Eur. J. Appl. Physiology 119 (8), 1701–1709. 10.1007/S00421-019-04160-3 31187282

[B20] Garcia-Retortillo S. Javierre C. Hristovski R. Ventura J. Balagué N. (2019b). Principal component analysis as a novel approach for cardiorespiratory exercise testing evaluation. Physiol. Meas. 40, 084002. 10.1088/1361-6579/ab2ca0 31239421

[B21] Garcia-Retortillo S. Rizzo R. Wang J. Sitges C. Ivanov P. C. (2020). Universal spectral profile and dynamic evolution of muscle activation: a hallmark of muscle type and physiological state. J. Appl. Physiology 129 (3), 419–441. 10.1152/japplphysiol.00385.2020 32673157 PMC7517426

[B22] Garcia-Retortillo S. Romero-Gómez C. Ivanov P. C. (2023). Network of muscle fibers activation facilitates inter-muscular coordination, adapts to fatigue and reflects muscle function. Commun. Biol. 6 (1), 891. 10.1038/s42003-023-05204-3 37648791 PMC10468525

[B23] Garcia-Retortillo S. Abenza Ó. Vasileva F. Balagué N. Hristovski R. Wells A. (2024). Age-related breakdown in networks of inter-muscular coordination. GeroScience 47, 1615–1639. 10.1007/s11357-024-01331-9 39287879 PMC11978574

[B24] Gohel V. Mehendale N. (2020). Review on electromyography signal acquisition and processing. Biophys. Rev. 12, 1361–1367. 10.1007/s12551-020-00770-w 33169207 PMC7755956

[B25] Hermens H. J. Freriks B. Disselhorst-Klug C. Rau G. (2000). Development of recommendations for SEMG sensors and sensor placement procedures. J. Electromyogr. Kinesiol. 10 (5), 361–374. 10.1016/s1050-6411(00)00027-4 11018445

[B26] Hunter S. Pereira X. Keenan K. (2016). Aging and exercise: the aging neuromuscular system and motor performance. J. Appl. Physiology 121 (4), 982–995. 10.1152/JAPPLPHYSIOL.00475.2016 27516536 PMC5142309

[B27] Ivanov P. C. (2021). The new field of network physiology: building the human physiolome. Front. Netw. Physiology 1, 711778. 10.3389/fnetp.2021.711778 36925582 PMC10013018

[B28] Ivanov P. C. Bartsch R. (2014). “Network physiology: mapping interactions between networks of physiologic networks,” in Networks of networks: the last frontier of complexity. Editors D’Agostino, G. Scala A. (Cham: Springer), 203–222. 10.1007/978-3-319-03518-5_10

[B29] Ivanov P. C. Liu K. Bartsch R. (2016). Focus on the emerging new fields of network physiology and network medicine. New J. Phys. 18, 100201. 10.1088/1367-2630/18/10/100201 30881198 PMC6415921

[B30] Ivanov P. C. Liu K. K. L. Lin A. Bartsch R. P. (2017). “Network physiology: from neural plasticity to organ network interactions,” in Emergent complexity from nonlinearity, in physics, engineering and the life sciences. Editors Mantica G. Stoop R. Stramaglia S. (Springer Proceedings in Physics), 191, 145–165.

[B31] Liu K. K. L. Bartsch R. P. Ma Q. D. Y. Ivanov P. C. (2015). Major component analysis of dynamic networks of physiologic organ interactions. J. Phys. Conf. Ser. 640 (1), 012013. 10.1088/1742-6596/640/1/012013 30174717 PMC6119077

[B32] Merletti R. Farina D. (2016). Surface electromyography: physiology, engineering and applications. Wiley-IEEE Press. 10.1002/9781119082934

[B33] Nicklas B. Brinkley T. Houston D. Lyles M. Hugenschmidt C. Beavers K. (2019). Effects of caloric restriction on cardiorespiratory fitness, fatigue, and disability responses to aerobic exercise in older adults with obesity: a randomized controlled trial. Journals Gerontology Ser. A 74 (7), 1084–1090. 10.1093/GERONA/GLY159 29982294 PMC6580693

[B34] Papagiannis G. Triantafyllou A. Roumpelakis I. Zampeli F. Garyfallia Eleni P. Koulouvaris P. (2019). Methodology of surface electromyography in gait analysis: review of the literature. J. Med. Eng. Technol. 43 (1), 59–65. 10.1080/03091902.2019.1609610 31074312

[B35] Prilutsky B. (2000). Coordination of Two- and one-joint muscles: functional consequences and implications for motor control. Mot. Control 4 (1), 1–44. 10.1123/mcj.4.1.1 10675807

[B36] Seidler R. Bernard J. Burutolu T. Fling B. Gordon M. Gwin J. (2010). Motor control and aging: links to age-related brain structural, functional, and biochemical effects. Neurosci. Biobehav. Rev. 34 (5), 721–733. 10.1016/J.NEUBIOREV.2009.10.005 19850077 PMC2838968

[B37] Serrien D. Swinnen S. Stelmach G. (2000). Age-related deterioration of coordinated interlimb behavior. Journals Gerontology Ser. B 55 (5), P295–P303. 10.1093/GERONB/55.5.P295 10985294

[B38] Sun W. Liang J. Yang Y. Wu Y. Yan T. Song R. (2016). Investigating aging-related changes in the coordination of agonist and antagonist muscles using fuzzy entropy and mutual information. Entropy 18 (6), 229. 10.3390/E18060229

[B39] Vecchio L. M. Meng Y. Xhima K. Lipsman N. Hamani C. Aubert I. (2018). The neuroprotective effects of exercise: maintaining a healthy brain throughout aging. Brain Plast. Amst. Neth. 4 (1), 17–52. 10.3233/BPL-180069 30564545 PMC6296262

[B40] Zampieri S. Pietrangelo L. Loefler S. Fruhmann H. Vogelauer M. Burggraf S. (2015). Lifelong physical exercise delays age-associated skeletal muscle decline. Journals Gerontology - Ser. A Biol. Sci. Med. Sci. 70 (2), 163–173. 10.1093/GERONA/GLU006 24550352

